# The Evolving Landscape of Systemic Therapy for Liposarcoma

**DOI:** 10.3390/cancers18111694

**Published:** 2026-05-22

**Authors:** Hee Kyung Kim, Akshat Sarkari, Warren A. Chow

**Affiliations:** 1 Chao Family Comprehensive Cancer Center, University of California, Orange, CA 92868, USA; heekyk2@hs.uci.edu (H.K.K.); asarkari@hs.uci.edu (A.S.); 2 Division of Hematology/Oncology, Department of Medicine, University of California, Orange, CA 92868, USA; 3 Department of Internal Medicine, Chungbuk National University Hospital, Cheongju 28644, Republic of Korea

**Keywords:** liposarcoma, systemic therapy, targeted therapy, MDM2 amplification, CDK4 inhibition, XPO1 inhibitor, T-cell receptor therapy

## Abstract

Background/Objectives: Liposarcoma represents a heterogeneous group of mesenchymal malignancies with distinct molecular profiles and clinical behaviors. While localized disease is managed with surgical resection, advanced or metastatic liposarcoma poses a significant therapeutic challenge due to limited response to traditional cytotoxic chemotherapy. This review summarizes current evidence-based systemic therapies and highlights recent advances in subtype-driven treatment strategies. Methods: We review key clinical trials supporting the use of anthracycline regimens, trabectedin, eribulin, and nuclear export inhibition with selinexor, as well as emerging targeted approaches directed at MDM2 and CDK4 amplification. In addition, we discuss the evolving role of immunotherapy, including checkpoint inhibitors and engineered T-cell receptor therapies targeting cancer–testis antigens. Results: Integrating molecular biology with therapeutic development, we emphasize the importance of histologic and genomic classification in guiding treatment selection and clinical trial design. Conclusion: Continued progress in biomarker-driven strategies and rational combination therapies is expected to further refine personalized treatment approaches and improve outcomes for patients with advanced liposarcoma.

## Simple Summary

Liposarcoma is a common type of soft tissue cancer, but treatment outcomes vary widely depending on the tumor subtype and genetic features. While traditional chemotherapy remains the standard treatment for advanced disease, many patients eventually experience progression, highlighting the need for improved therapies. This review summarizes current systemic treatment approaches and examines emerging strategies based on the molecular biology of liposarcoma. We discuss recently approved drugs, targeted therapies aimed at specific genetic alterations, and new immunotherapy approaches, including engineered T-cell treatments. By integrating clinical trial results with advances in tumor biology, this review aims to clarify how subtype-specific treatment selection and biomarker-guided strategies may improve outcomes and guide future research directions in liposarcoma.

## 1. Introduction

Liposarcoma (LPS) is the most common subtype of soft tissue sarcoma, accounting for 15–20% of all soft tissue sarcoma [1]. LPS comprises malignant tumors originating from adipose tissue and subtypes are categorized based on cell morphology, immunohistochemistry and genetic alterations [2,3]. The median age at diagnosis of LPS is 50–60 years old with a slight male predominance [4,5]. The anatomic distribution varies by histologic subtypes: well-differentiated and dedifferentiated liposarcomas occur predominantly in the retroperitoneum, accounting for over 50% of retroperitoneal sarcomas, while myxoid and pleomorphic liposarcomas more often arise in the extremities [6,7].

LPS is classified into five subtypes according to the 2020 World Health Organization Classification of Tumors of Soft Tissue and Bone: atypical lipomatous tumor/well-differentiated liposarcoma (ALT/WDLPS), dedifferentiated liposarcoma (DDLPS), myxoid liposarcoma (MLPS), pleomorphic liposarcoma (PLPS), and myxoid pleomorphic liposarcoma (MPLPS). Each subtype demonstrates distinctive clinical presentations and biologic behavior, including genetic mutations, recurrence rates, and metastatic potential [8].

Treatment of LPS requires a multidisciplinary approach and varies by subtype and disease stage. For localized disease, complete surgical resection is the cornerstone of treatment [9], whereas systemic therapy is primarily used for unresectable, recurrent, or metastatic settings and is increasingly tailored to histology and clinical context [10]. At the same time, expanding molecular insights, such as recurrent pathway dependencies and subtype-defining genomic events, have accelerated the development of targeted and immune-based strategies. These strategies aim to move beyond traditional cytotoxic chemotherapy and enable biology-driven treatment selection.

In this review, we summarize the current standard management of LPS and provide a comprehensive overview of newly approved therapies as well as emerging treatment strategies and investigational agents under active clinical and translational research.

## 2. Current Standard of Systemic Treatment for Liposarcoma

An overview of these subtype-specific molecular features and systemic treatment implications is summarized in Table 1. Before discussing emerging therapies for advanced LPS, it is important to delineate current evidence-based treatment, which varies by histologic subtypes and clinical setting. For localized diseases, surgery remains the primary treatment option for all subtypes. However, for unresectable, recurrent, or metastatic disease, systemic therapy plays a central role as outlined by the National Comprehensive Cancer Network (NCCN) [11]. For unresectable disease, anthracycline-based chemotherapies have historically been preferred, including doxorubicin (or epirubicin) as monotherapy or combination regimens such as AIM (doxorubicin, ifosfamide, mesna) [11,12]. Gemcitabine-based regimens (gemcitabine alone or in combination with docetaxel) are also recommended and are frequently used depending on patient fitness and prior therapy [11].

These treatment guidelines were established through multiple studies. For example, the EORTC 62012 trial compared doxorubicin vs. doxorubicin-ifosfamide in 455 patients with metastatic, locally advanced, or unresectable soft tissue sarcoma. While there was no significant difference in overall survival(OS) between the two groups (median OS 12.8 months versus 14.3 months, HR 0.83, *p* = 0.076), progression-free survival(PFS) was significantly higher in the doxorubicin plus ifosfamide group (7.4 months versus 4.6 months, HR 0.74, *p* = 0.003) with an objective response rate(ORR) of 26% versus 14% (*p* < 0.0006) [13,14]. However, rates of toxicity were expectedly higher in the combination group, with grade 3–4 febrile neutropenia occurring in 46% versus 13% of patients [13]. Subgroup analyses suggested that patients with high-grade tumors, including undifferentiated pleomorphic sarcoma (UPS), may be more likely to benefit from combination therapy in terms of tumor response [15].

Importantly, chemosensitivity differs by histologic subtype. While MLPS has historically demonstrated better responsiveness to cytotoxic therapy, WDLPS and DDLPS are relatively chemo-insensitive [16,17]. However, retrospective single-institution data suggest that a subset of patients with retroperitoneal DDLPS may derive modest but clinically meaningful disease control from anthracycline-based combination chemotherapy. Livingston et al. [18] reported results from MD Anderson Cancer Center in 82 patients with retroperitoneal WD/DDLPS treated with first-line chemotherapy. Among these patients, the clinical benefit rate was 61% (partial response 21%, stable disease 40%), with median OS of 29 months from the start of chemotherapy. Notably, all RECIST responses were observed in patients receiving combination chemotherapy rather than single-agent therapy [18].

For subsequent lines of therapy, trabectedin is supported by Category 1 evidence for LPS in the NCCN guidelines [11]. Trabectedin binds to the minor groove of DNA, which affects the function of DNA-binding proteins including transcription factors and DNA repair machinery, resulting in perturbation of the cell cycle and induction of p53-independent apoptosis [19,20] A phase III multicenter trial by Demetri et al. [19] demonstrated superior PFS compared with dacarbazine in 518 patients with advanced LPS/leiomyosarcoma (median PFS 4.2 versus 1.5 months, HR 0.55, *p* < 0.001), with 3-month and 6-month progression-free rates of 56% versus 34% and 37% versus 14%, respectively. Although no significant difference in OS was observed in the primary analysis, the safety profiles of both treatment modalities were manageable [19,21].

Importantly, clinical activity of trabectedin appears to be more pronounced in MLPS than in other histological subtypes [22]. This observation may be related to the subtype-defining FUS::DDIT3 fusion, as preclinical studies suggest that trabectedin can disrupt fusion-driven transcriptional programs and promote adipocytic differentiation, consistent with its activity in translocation-associated sarcomas [23,24].

In addition to trabectedin, eribulin has emerged as an important later-line option for LPS and is also supported as a Category 1 regimen in the NCCN guidelines [11]. Eribulin, a microtubule dynamics inhibitor, demonstrated an OS over dacarbazine in a multicenter phase III study of 452 previously treated patients with advanced LPS or leiomyosarcoma (median OS 13.5 versus 11.5 months, HR 0.77, *p* = 0.0169) [25].

In the LPS subgroup specifically (n = 143), the survival benefit was particularly pronounced, with median OS of 15.6 versus 8.4 months (HR 0.51, *p* < 0.001) and median PFS of 2.9 versus 1.7 months (HR 0.52, *p* = 0.0015) [26]. In the histology-specific subgroup analysis from the same trial, the OS benefit with eribulin was consistently observed across all LPS subtypes, with HRs favoring eribulin over dacarbazine in dedifferentiated (HR 0.69; 95% CI, 0.36–1.33), myxoid/round cell (HR 0.57; 95% CI, 0.29–1.11), and pleomorphic LPS (HR 0.34; 95% CI, 0.09–1.30), although individual subtype analyses were underpowered to achieve statistical significance [26]. Grade ≥3 adverse events were more frequent with eribulin (67%) than with dacarbazine (56%) [25]. Collectively, these data establish trabectedin and eribulin as evidence-based standards beyond anthracycline-based therapy for advanced disease.

**Figure 1:**
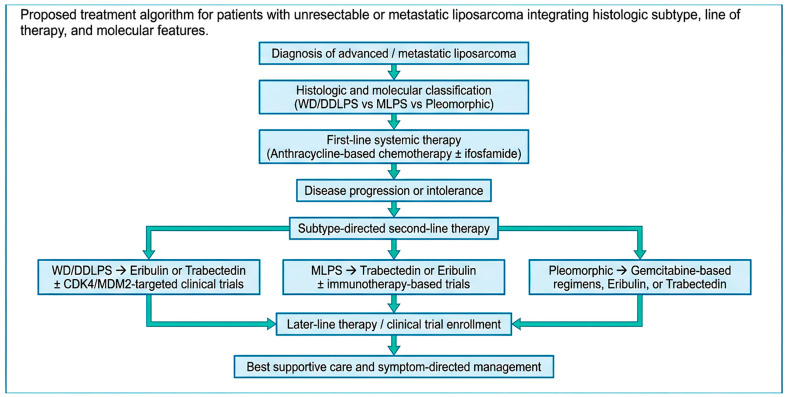
Systemic treatment algorithm for advanced liposarcoma. Created by the authors using Gemini (Google).

Beyond these agents, pazopanib is listed as a later-line option in NCCN guidance; however, its activity is better established in non-adipocytic soft tissue sarcomas. The pivotal PALETTE trial demonstrated improved PFS with pazopanib versus placebo in 369 patients with metastatic non-adipocytic soft tissue sarcoma (median PFS 4.6 versus 1.6 months, *p* < 0.0001), while specifically excluding adipocytic sarcomas from enrollment based on limited activity observed in a prior phase II study [27,28], Pazopanib therefore remains primarily an option for non-adipocytic sarcoma subtypes [11,27]. Key pivotal clinical trials supporting current standard systemic therapies in advanced liposarcoma are summarized in Table 2. Taken together, we propose a practical systemic treatment algorithm for advanced LPS that integrates histologic subtype, line of therapy, and molecular features (Figure 1).

## 3. Targeting the MDM2-CDK4 Axis in WDLPS/DDLPS

Regarding DDLPS and WDLPS, much has been explored regarding the efficacy of standard-of-care treatment as described above. However, recent exploratory advances have been made in targeted therapy for LPS through careful examination of gene/chromosomal loci and proteins involved in propagation of LPS. Specifically, both WDLPS and DDLPS are known to have amplification of the chromosome 12q13-15 region. In this specific regions, two expressible genes predominate: MDM2 and CDK4 [29]. These two genes are well-described to be oncogenes as they are involved in a number of other cancers, highlighting their potential important role in LPS tumor propagation. MDM2 functions as an E3 ubiquitin ligase that promotes ubiquitination and proteasomal degradation of the p53 tumor suppressor protein [30,31]. CDK4, in complex with cyclin D, facilitates G1–S cell-cycle progression, in part through phosphorylation of RB [32,33]. In well-differentiated and dedifferentiated LPS, amplification of MDM2 and CDK4 is frequent and has provided a strong rationale to explore these pathways as therapeutic targets [34,35]. Accordingly, MDM2 inhibition is intended to restore p53 pathway activity in tumors with intact TP53, whereas CDK4/6 inhibition aims to suppress cell-cycle progression in CDK4-driven disease [32,34].

### 3.1. MDM2 Inhibition

One of the first studies to illustrate the proof-of-concept mechanism of MDM2 inhibition was by Ray-Coquard et al. [36] who utilized a small-molecule MDM2 antagonist (RG7112) in previously untreated patients with WDLPS or DDLPS. Fascinatingly, the investigators found that by the end of the treatment period with RG7112 as compared to baseline, patients who received treatment with RG7112 was associated with a median 4.86-fold elevation in p53 concentration compared to baseline. One patient had a partial response though the other 14 patients were noted to have stable disease (out of 20 total enrolled) [36]. While these results were certainly preliminary and did not overall alter patient outcomes, it suggested that preservation of p53 function was possible in both WDLPS and DDLPS.

More contemporary MDM2–p53 antagonists have been developed to improve potency, pharmacokinetics, and tolerability compared with first-generation agents. The phase III MANTRA trial investigated milademetan (RAIN-32) against trabectedin in patients with unresectable/metastatic DDLPS. However, the trial failed to meet its primary endpoint, with median PFS of 3.6 months for milademetan versus 2.2 months for trabectedin (hazard ratio 0.89, 95% CI 0.61–1.29, *p* = 0.53) [37,38]. Despite promising early-phase data, these results highlighted the challenges of translating target engagement into durable clinical efficacy. The most common grade 3/4 treatment-emergent adverse events with milademetan included thrombocytopenia (39.5%), neutropenia (25.5%), and anemia (18.6%), with 44.2% of patients requiring dose reductions [39]. Based on these disappointing results, further development of milademetan in DDLPS was discontinued.

In contrast, brigimadlin (BI 907828) has shown encouraging early clinical activity in DDLPS. In a phase Ia/Ib study of 90 patients with DDLPS receiving brigimadlin 45 mg once every 3 weeks, the median PFS was 8.1 months with an ORR of 18.6% (1 complete response and 15 partial responses) and a disease control rate of 88.4% [40]. Grade ≥3 treatment-related adverse events occurred in 42.9% of patients, most commonly thrombocytopenia (22.6%), neutropenia (22.6%), and anemia (9.5%), with only 4.8% of patients discontinuing due to adverse events [40]. Based on these encouraging preliminary data, brigimadlin was granted Fast Track Designation by the FDA, prompting the initiation of the phase II/III Brightline-1 trial (NCT05218499) to compare brigimadlin with doxorubicin as first-line therapy in advanced DDLPS [41]. However, at the 2024 Connective Tissue Oncology Society (CTOS) annual meeting, it was announced that the Brightline-1 trial failed to demonstrate superiority over doxorubicin, leading to the subsequent discontinuation of brigimadlin’s clinical development across all indications [42]. As a result, further recruitment and evaluation in the single-arm phase III Brightline-4 trial (NCT06058793) were also halted [43]. These developments underscore an ongoing shift toward biomarker-enriched, subtype-focused trials and may inform rational combinations with CDK4/6 inhibition or other pathway-directed approaches [44].

### 3.2. CDK 4/6 Inhibition

More than 90% of WDLPS/DDLPS contain CDK4 amplification [29]. Accordingly, CDK4 has emerged as a potential therapeutic target, similar to MDM2. Indeed, palbociclib has been shown to prevent growth of CDK4-amplified LPS cell lines in xenograft models [45]. Dickson et al. conducted a phase II trial of the CDK4 inhibitor palbociclib in patients with advanced WDLPS/DDLPS treated with 125 mg once daily for 21 days for 12 cycles. Of 28 patients treated compared to placebo, 14 were progression-free at 12 weeks, representing a 12-week PFS rate of 50% [46]. In addition, there was one complete response, which lasted over 2 years. Importantly, only 3 patients required dose reduction due to adverse events. The results of the study showed promise in acknowledging CDK4 as a viable therapeutic target for patients with advanced DDLPS/WDLPS. Clinical outcomes from CDK4/6 inhibitor studies in DDLPS, including palbociclib monotherapy, are summarized in Table 3.

Abemaciclib is a newer CDK4/6 inhibitor with continuous dosing and has been evaluated in progressive DDLPS. In a phase II study, abemaciclib achieved a 12-week progression-free rate of 76% with a median PFS of approximately 30 weeks (7.0 months), supporting ongoing randomized evaluation [47,56]. Mechanistic correlates suggest that therapy-induced senescence may contribute to clinical benefit, providing a rationale for combination strategies designed to eliminate senescent tumor cells or prevent senescence escape [47,56]. The phase III SARC041 trial (NCT04967521) is currently evaluating abemaciclib 200 mg PO twice daily versus placebo in patients with advanced DDLPS, with crossover allowed upon progression [48,49]. This randomized, double-blind study has completed accrual with a target enrollment of 108 evaluable patients and PFS as the primary endpoint, representing a critical step in establishing CDK4/6 inhibition as standard therapy in DDLPS [3,49]. The results of the phase III SARC041 trial are anticipated to be presented at ASCO annual meeting 2026 [48].

### 3.3. Combination Strategies

Recent studies have attempted to evaluate synergistic effects of both MDM2 and CDK4 inhibition. Larcohe-Clary et al. [57] evaluated this from the preclinical standpoint, examining signaling/survival pathway changes in DDLPS cells treated with the MDM2 antagonist, RG7388 (idasanutlin) and the CDK4 inhibitor, palbociclib. Overall, the authors found that the combination of RG7388 and palbociclib significantly increased apoptosis above anti-tumor effects of either agent alone [57]. In a xenograft model, tumor growth rate was significantly reduced with both agents as compared to either agent alone, and median PFS was significantly increased with combination therapy [57]. These findings suggested a favorable interaction between CDK4 inhibitors and agents affecting MDM2 expression/function, as treatment with palbociclib was associated with post-translational downregulation of MDM2, additionally lower MDM2 expression in tumor biopsies after palbociclib treatment correlated with tumor response [34].

Based on preclinical observations, clinical evaluation of CDK4/6 inhibition combined with PD-1 blockade is ongoing. A phase II trial (NCT04438824) is examining palbociclib (125 mg once daily orally for 21 days followed by 7 days off) combined with the anti-PD-1 inhibitor retifanlimab (500 mg IV flat dose every 4 weeks) in up to 30 patients with recurrent, unresectable, or metastatic DDLPS [50]. This combination was designed based on preclinical evidence that CDK4/6 inhibitors upregulate antigen processing and presentation, suppress regulatory T cells, and increase inflammation within the tumor microenvironment, potentially synergizing with anti-PD-1 blockade [49,57]. Alternative combination approaches, including ribociclib with siremadlin, have been explored but showed limited activity with dose-limiting hematologic toxicities [34].

## 4. Targeting Nuclear Export: The Role of XPO1 Inhibitors

### 4.1. Mechanism of XPO1 Inhibition in DDLPS

Targeting nuclear export with selinexor represents an investigational therapeutic approach for DDLPS [58]. Selinexor is a first-generation oral selective inhibitor of nuclear export (SINE) that targets exportin-1 (XPO1), which is overexpressed in LPS [59]. XPO1 is the primary nuclear exporter for over 200 cargo proteins, including key tumor suppressor proteins (TSPs) such as p53, p21, and p27, as well as the glucocorticoid receptor (GR). Overexpression of XPO1 leads to excessive nuclear export of cargo proteins, resulting in their functional inactivation. Selinexor, a selective inhibitor of SINE, binds covalently to the cysteine-528 of XPO1. By inhibiting XPO1, selinexor promotes nuclear retention of tumor suppressor proteins like p53, IGFBP5, and IκB, while also downregulating oncogenic proteins such as aurora kinases A and B [60,61].

In DDLPS, which frequently shows MDM2 and CDK4 amplifications, Selinexor induces cell cycle arrest and apoptosis independently of traditional pathways involving p53 and RB1 [62]. In addition, selinexor has been shown to induce PARP cleavage, a key marker of apoptosis, in various sarcoma models [63]. This mechanism induces apoptosis and inhibits tumor growth, particularly in MDM2- and CDK4-amplified DDLPS [59].

### 4.2. Clinical Development and Efficacy of Selinexor

In a phase Ib study, selinexor monotherapy resulted in durable stable disease (≥4 months) in 47% of evaluable patients with DDLPS. Consistent with these findings, earlier-phase analyses suggested that the therapeutic activity of selinexor was more pronounced in DDLPS compared with well-differentiated disease [64]. The pivotal phase II/III SEAL trial (NCT02606461) evaluated selinexor versus placebo in patients with advanced, previously treated DDLPS. In this placebo-controlled, double-blind, randomized trial enrolling patients with DDLPS refractory to prior systemic therapies, selinexor achieved its primary endpoint of PFS improvement, with a median PFS of 2.8 months versus 2.1 months in the placebo arm (HR 0.70; *p* = 0.0228). The PFS benefit conferred by selinexor was generally consistent across prespecified subgroups evaluated in the SEAL trial. However, no significant difference in OS was observed between the selinexor and placebo arms. Furthermore, the clinical utility of selinexor has been challenged by its characteristic toxicity profile, which includes dose-limiting gastrointestinal adverse events (e.g., nausea, anorexia) and significant constitutional symptoms (e.g., fatigue). These findings underscore the critical importance of proactive supportive care and timely dose modifications to optimize tolerability and, ultimately, preserve the patient’s quality of life during treatment [52]. Key clinical data for XPO1 inhibition in DDLPS, including the phase III SEAL trial of selinexor, are summarized in Table 3.

### 4.3. Combination Strategies and Next-Generation SINE Compounds

To enhance efficacy and overcome potential resistance mechanisms, research has pivoted toward rational combination strategies and the development of next-generation SINE compounds. The combination of selinexor with doxorubicin is an actively investigated regimen. This pairing is designed to leverage the synergistic effect of XPO1 inhibition, which prevents the repair of doxorubicin-induced DNA double-strand breaks, thereby potentially enhancing efficacy in DDLPS. An initial Phase Ib study (NCT03042819) involving 25 patients with various soft tissue sarcomas demonstrated promise, yielding an overall ORR of 21% and a median PFS of 5.5 months across all subtypes. However, this combination was associated with considerable hematologic toxicity, notably neutropenia (56%) and febrile neutropenia (28%), highlighting the need for careful dose management [53].

Parallel efforts focus on improving the therapeutic index. Eltanexor (KPT-8602), a second-generation SINE compound, was designed with lower blood–brain barrier penetration compared to selinexor. Preclinical and early-phase studies suggest that eltanexor maintains potent antitumor activity while exhibiting a significantly improved tolerability profile, particularly concerning centrally mediated side effects such as anorexia and fatigue. This improved profile positions eltanexor as a promising candidate for long-term maintenance strategies in LPS [65].

## 5. Immunotherapy and Cellular Therapies

### 5.1. TCR-T Cell Therapy

Initial findings examining the efficacy of protein inhibitors in DDLPS and WDLPS paved the way for exploration of targeted immunotherapy, especially for advanced treatment of refractory sarcomas. T-cell receptor gene-modified therapy (TCR-T) represents a transformative approach in LPS treatment, particularly targeting the cancer/testis antigen NY-ESO-1. The NY-ESO-1 antigen is expressed in 80–90% of patients with metastatic MLPS, formerly known as myxoid/round cell liposarcoma (MRCLS), making it an attractive therapeutic target [54,66,67].

Early TCR-T development was pioneered by Ishihara et al. [67], who established preclinical relevance in a mouse sarcoma model with resistance to immune checkpoint inhibition, followed by a phase I clinical trial in 3 patients with refractory synovial sarcoma, which demonstrated distinct tumor shrinkage despite the occurrence of cytokine release syndrome in two patients [67].

In August 2024, afamitresgene autoleucel (afami-cel; TECELRA) received FDA accelerated approval for adults with unresectable or metastatic synovial sarcoma who had received prior chemotherapy, representing the first FDA-approved T-cell receptor gene therapy for any cancer [54,55]. Of particular relevance to LPS, afami-cel targets MAGE-A4, an antigen that is expressed not only in synovial sarcoma but also in MLPS. This shared antigen expression raises the possibility that TCR-T cell therapy may have therapeutic applicability beyond synovial sarcoma. However, eligibility requires patients to carry specific HLA-A*02 alleles, which may limit the proportion of LPS patients who are candidates for this approach [55]. The encouraging efficacy and survival outcomes observed in SPEARHEAD-1 provide a compelling rationale for dedicated investigation of afami-cel and related TCR-T strategies in MAGE-A4-expressing LPS subtypes, particularly MLPS, and clinical trials in this population are warranted.

More recently, clinical development has expanded to include NY-ESO-1-directed TCR therapies specifically in MLPS. Letetresgene autoleucel (lete-cel; NY-ESO-1c259T), another NY-ESO-1-targeted TCR-T therapy, has shown promising activity in patients with HLA-A*02-positive, NY-ESO-1-expressing advanced/metastatic MLPS [68]. In the pivotal phase II IGNYTE-ESO trial, lete-cel demonstrated an overall response rate of 43% in MLPS (13/30 patients), with a median duration of response of 12.2 months [54,69]. Based on these results, lete-cel received FDA breakthrough therapy designation in January 2025 for patients with unresectable or metastatic MLPS who have received prior anthracycline-based chemotherapy [69]. Safety findings showed that all patients experienced treatment-emergent adverse events, with cytopenias, cytokine release syndrome, and rash being the most common, though overall toxicities were manageable [70].

### 5.2. Immune Checkpoint Inhibitors

The therapeutic potential of immune checkpoint inhibitors has been investigated across a broad spectrum of sarcomas, including both soft-tissue and bone subtypes. The SARC028 trial, a phase II multicenter study, evaluated pembrolizumab in advanced soft tissue sarcoma and bone sarcoma [71]. Between March 2015 and February 2016, 86 patients were enrolled, with 80 evaluable for responses. Seven (18%) of 40 patients with soft-tissue sarcoma had an objective response, including two (20%) of ten patients with LPS [71,72]. The median PFS was 18 weeks for soft tissue sarcoma patients, with responses limited primarily to patients with UPS and DDLPS [71,73]. Although the primary endpoint was not met, pembrolizumab showed promising activity in these specific sarcoma subtypes. Correlative analyses revealed that patients who responded to pembrolizumab were more likely to have higher densities of activated T cells (CD8+ CD3+ PD-1+) and increased percentage of tumor-associated macrophages expressing PD-L1 at baseline compared with non-responders [72]. Additionally, the PEMBROSARC trial further underscored the significance of tertiary lymphoid structures (TLS) as a key predictive biomarker for treatment response. In this study, the presence of TLS within the tumor microenvironment was associated with significantly improved objective response rates and progression-free survival in patients treated with pembrolizumab [74].

In addition to pembrolizumab, nivolumab and ipilimumab have been explored as immunotherapeutic options for patients with metastatic sarcoma. A multicenter phase II study conducted by Seligson et al. evaluated patients with metastatic sarcoma who were randomized to nivolumab versus nivolumab plus ipilmumab [75]. Expansion cohorts were subsequently enrolled to further evaluate specific histologies. In the expansion cohorts including 66 patients, the DDLPS cohort (n = 24) met the primary endpoint in the nivolumab plus ipilimumab group [75,76]. Specifically, the overall response rate with nivolumab plus ipilimumab was 14.3% in patients with DDLPS versus 6.7% with nivolumab alone. In the DDLPS cohort, the median PFS was 4.6 months with nivolumab alone versus 5.5 months with the combination, and median OS was 8.1 months with nivolumab versus 13.1 months with the combination [75].

A neoadjuvant approach was also explored in the NCT03307616 trial, which randomized patients with resectable retroperitoneal DDLPS (n = 17) and extremity/truncal UPS (n = 10) to neoadjuvant nivolumab or nivolumab plus ipilimumab [77]. Pathologic response, assessed by percent hyalinization, differed substantially between histological subtypes, with a median of 8.8% in DDLPS compared with 89% in UPS [77]. Lower densities of regulatory T cells before treatment were associated with major pathologic response, and multi-omic correlative analyses revealed that DDLPS tumors had lower baseline immune infiltration and reduced T cell activation compared to UPS [77,78]. These findings suggest differential immune biology between DDLPS and UPS that may inform future therapeutic strategies and highlight the importance of histology-specific trial design in sarcoma immunotherapy.

Overall, these studies implicate nivolumab and ipilimumab combination therapy as an emerging therapeutic alternative for patients with metastatic sarcoma, particularly in select histologic subtypes such as DDLPS and UPS, though further research is needed to identify predictive biomarkers and optimize patient selection [75,79]. Selected ongoing and investigational clinical trials across targeted, cell-cycle, nuclear export, and immunotherapeutic strategies are summarized in Table 3.

## 6. Other Emerging Therapeutic Strategies

Beyond MDM2 and CDK4/6 targeting, several additional therapeutic avenues are under active investigation in LPS, largely driven by recurrent pathway alterations, tumor microenvironment features, and treatment resistance mechanisms identified in contemporary genomic and epigenomic profiling studies. These strategies include multi-targeted kinase inhibition, nuclear export inhibition, and epigenetic modulation.

### 6.1. Multi-Targeted Kinase Inhibitors and Anti-Angiogenic Strategies

Anti-angiogenic approaches have shown variable activity across soft-tissue sarcoma, and LPS has generally been less sensitive to single-agent VEGFR inhibition. This is exemplified by the randomized, double-blind, placebo-controlled phase II SARC024 trial, in which regorafenib failed to improve PFS over placebo in 48 patients with treatment-refractory LPS (median PFS 1.87 vs. 2.07 months; HR 0.85, *p* = 0.62), with no objective responses observed in the regorafenib arm [80]. However, broader-spectrum receptor tyrosine kinase inhibitors may provide disease control in selected WDLPS/DDLPS. In a phase II study of sitravatinib (MGCD516), a multi-kinase inhibitor targeting RTKs including MET, AXL, and VEGFR family members, the primary endpoint was met with 41% of patients (12/29) remaining alive and without disease progression at 12 weeks, although no confirmed objective responses were observed. Median PFS and OS were 11.7 weeks and 31.7 weeks, respectively [81,82]. The majority of treatment-related adverse events were grade 1–2 in severity, with the most frequently reported events being diarrhea (59%), hypertension (52%), hoarseness (41%), mucositis (31%), and nausea (31%) [82].

Anlotinib, a multi-targeted TKI inhibiting VEGFR, FGFR, PDGFR, and c-Kit, has been evaluated in soft-tissue sarcoma populations including LPS, particularly in Chinese clinical trials. In a phase II trial of first-line anlotinib for chemotherapy-ineligible patients with advanced soft tissue sarcoma (NCT03792542), 40 patients were enrolled, including 10 with LPS. The median PFS was 6.83 months overall, with the LPS subgroup achieving a median PFS of 8.71 months and median OS of 16.23 months [83]. In the LPS subgroup, first-line anlotinib showed numerically longer outcomes than in later-line settings reported previously, with a median PFS of 5.6 months and median OS of 13.0 months [84]. Another trial evaluated anlotinib as maintenance treatment after first-line chemotherapy in 49 patients, including 17 with LPS (35%). The median PFS of anlotinib maintenance was 11.3 months, with improved survival observed in LPS, synovial sarcoma, and leiomyosarcoma [85,86]. However, these studies remain primarily regional, and further prospective, subtype-enriched studies are needed to define benefit and optimal sequencing in LPS globally.

### 6.2. Autophagy Modulation and Metabolic Targeting

Nuclear export inhibition with selinexor and eltanexor is discussed in detail in Section 4. Beyond XPO1 inhibition, targeting autophagy has emerged as a promising strategy in LPS. Autophagy is a cellular recycling process that supports tumor cell survival under stress and can be modulated through the mTOR pathway.

In patient-derived orthotopic xenograft models of DDLPS, combined targeting of mTOR and autophagy pathways using rapamycin and chloroquine, respectively, yielded synergistic antitumor activity, evidenced by a substantial increase in apoptosis among cancer cells [87]. Further investigation in WDLPS cell lines revealed that the combination of rapamycin and chloroquine led to overproduction of autophagosomes, which resulted in extensive apoptosis. The combination significantly inhibited cell viability more than either drug alone [88]. Phase II studies of the mTOR inhibitor ridaforolimus showed a 6-month PFS rate of 23.4% in soft tissue and bone sarcomas, including LPS [89], though a subsequent phase III trial demonstrated only modest benefit [90].

The rationale for targeting autophagy in LPS stems from aberrant mTOR activation observed in these tumors [91,92]. mTOR negatively regulates autophagy, and its inhibition induces autophagy as a survival mechanism. Paradoxically, blocking autophagy with chloroquine while simultaneously inducing it with mTOR inhibitors creates a therapeutic vulnerability by causing accumulation of dysfunctional autophagosomes and cellular stress [88]. This dual-targeting strategy represents a promising preclinical hypothesis that may address resistance, though clinical efficacy remains to be established in human trials.

### 6.3. Epigenetic and Transcriptional Therapies

Aberrant epigenetic regulation, encompassing methylation of DNA, modification of histones, chromatin restructuring, and dysregulation of non-coding RNAs, plays a pivotal role in LPS initiation and progression across subtypes, representing potentially druggable vulnerabilities. In MLPS, FUS::DDIT3 fusion-driven transcriptional reprogramming governs adipocytic differentiation, and the transition to round cell morphology has been linked to transcriptional suppression of the DLK1-DIO3 locus at 14q32, with consequent upregulation of YY1, C-MYC, and HDAC2, collectively facilitating accelerated cell cycle progression [93,94]. These findings suggest trabectedin’s efficacy in MLPS may relate to its effects on fusion-driven transcription.

In WDLPS/DDLPS, epigenetic alterations and histone deacetylase (HDAC) dependencies have prompted interest in HDAC inhibitors. Preclinical studies identified HDAC2 as highly co-expressed with MDM2 in DDLPS, with elevated HDAC2 expression associated with worse disease-free survival. Treatment with HDAC inhibitors MI-192 (HDAC2/3 inhibitor) or romidepsin (HDAC1/2 inhibitor) reduced MDM2 expression and induced apoptosis in DDLPS cell lines, with romidepsin reducing tumor growth in murine xenograft models [95]. Additionally, the class I/II HDAC inhibitor givinostat combined with doxorubicin showed significant potential in improving sensitization in preclinical models of various sarcomas including LPS [96].

However, clinical translation has been challenging, as medications that alter DNA methylation status, such as HDAC inhibitors, have provided only modest clinical benefit in soft-tissue sarcomas in early-phase clinical trials despite efficacy in hematologic malignancies [3,51]. A key challenge is identifying predictive biomarkers and managing overlapping hematologic toxicities when combining epigenetic agents with cytotoxic or cell-cycle-directed therapies. Further investigation is needed to characterize potential interactions between MDM2 hyperfunction and epigenetic modifications in DDLPS and to develop rational biomarker-driven combination strategies.

### 6.4. Rational Combinations to Overcome Resistance

Given the limited depth and duration of response with most single agents, combination strategies are a major focus. Prominent examples include dual targeting of MDM2 and CDK4/6, combinations of CDK4/6 inhibitors with anti-PD-1 therapy (as discussed in Section 5), autophagy modulation with mTOR inhibition, and combinations of targeted agents with chemotherapy to improve response rates without prohibitive toxicity. However, early clinical attempts at combined MDM2 and CDK4/6 inhibition have encountered dose-limiting hematologic toxicities, emphasizing the need for careful dose optimization and patient selection [97]. Future trials will likely require integrated correlative studies to define pharmacodynamic effects, mechanisms of escape, and optimal patient selection to maximize therapeutic benefit while minimizing toxicity.

## 7. Future Directions and Clinical Challenges

Future progress in LPS will depend on aligning therapy with subtype-specific biology while addressing practical barriers in clinical trial design for rare cancers. MDM2 and CDK4 amplifications are well-established diagnostic markers in LPS, and molecular profiling of these alterations is essential for treatment decisions and facilitating targeted therapies [98,99]. Tumor genotyping using next-generation sequencing on formalin-fixed paraffin-embedded samples has become increasingly common in clinical practice, offering the hope of personalized targeted therapy and identifying novel targets [100]. Key needs include standardized molecular profiling (MDM2/CDK4 amplification testing for WDLPS/DDLPS via fluorescence in situ hybridization or immunohistochemistry; fusion testing for MLPS), harmonized response assessment beyond RECIST where appropriate, and systematic collection of pretreatment and on-treatment biospecimens.

### 7.1. Biomarker-Driven and Subtype-Enriched Trials

Because LPS subtypes differ in genomic drivers, immune contexture, and chemosensitivity, pooling across histologies can dilute signals of activity. Enrichment based on molecular features (e.g., MDM2 amplification for MDM2–p53 antagonists; NY-ESO-1 expression and HLA-A*02 type for TCR therapies) is increasingly feasible and should be prioritized. The STRASS2 trial (EORTC 1809), a phase III multicenter international trial, demonstrates this approach by randomizing 250 patients with high-grade retroperitoneal DDLPS or leiomyosarcoma (stratified based on histology) to receive three cycles of neoadjuvant anthracycline-based chemotherapy followed by surgery or surgery alone, with disease-free survival as the primary outcome [101,102]. This histology-specific design recognizes that high-grade DDLPS and leiomyosarcoma have distinct biology and recurrence patterns that warrant tailored therapeutic approaches.

### 7.2. Integrating Local and Systemic Therapy

For retroperitoneal WDLPS/DDLPS, durable local control remains central, with complete macroscopic resection as the cornerstone of management [103,104]. Systemic therapy is considered selectively to complement surgery, particularly when tumor biology or anatomic constraints suggest a high risk of recurrence or when downstaging could facilitate resection. Neoadjuvant approaches, including immune checkpoint blockade as discussed in Section 5, are being explored to enable biologic response assessment and facilitate translational endpoints, though responses in DDLPS have been modest compared to other sarcoma subtypes [77,78].

For high-risk extremity and trunk wall sarcomas, the role of neoadjuvant chemotherapy has been evaluated in randomized trials. The ISG-STS 1001 phase III trial by Gronchi et al. [105] compared standard neoadjuvant chemotherapy (epirubicin plus ifosfamide for three cycles) versus histotype-tailored chemotherapy in 286 patients with high-risk soft tissue sarcoma, including high-grade MLPS, treated with trabectedin in the histotype-tailored arm), leiomyosarcoma, synovial sarcoma, malignant peripheral nerve sheath tumor, and UPS [105]. Overall, across all subtypes, there was no benefit of histotype-tailored chemotherapy over standard chemotherapy, with projected 46-month disease-free survival of 62% for standard versus 38% for histotype-tailored therapy [105]. However, the expanded high-grade cohort (n = 101) demonstrated that trabectedin was non-inferior to anthracycline plus ifosfamide in this specific subtype, with 5-year disease-free survival of 86% versus 73% (hazard ratio 0.72, 95% CI 0.36–1.43) and a more favorable toxicity profile [106]. These findings support the use of anthracycline-based regimens when neoadjuvant chemotherapy is considered for high-risk extremity or trunk wall sarcomas, and they suggest that trabectedin may be a reasonable alternative for selected patients with high-grade MLPS. Furthermore, the results of the phase II SU2C-SARC032 trial (Mowery et al., 2024 [107]) have introduced a practice-changing approach for other aggressive subtypes. The study demonstrated that adding perioperative pembrolizumab to preoperative radiotherapy and surgery significantly improved 2-year disease-free survival (67% vs. 52%; HR 0.61) in patients with stage III dedifferentiated or pleomorphic liposarcoma of the extremity. These findings establish the integration of immune checkpoint blockade as a new standard-of-care option for these specific high-risk histologies [107].

For retroperitoneal DDLPS, surgical resection often requires concomitant nephrectomy, and nephrectomy is associated with worse short-term postoperative renal function [108]. When systemic therapy is being considered, a neoadjuvant approach may be reasonable to avoid postoperative renal impairment that could limit the feasibility or intensity of subsequent systemic treatment. More broadly, neoadjuvant or perioperative systemic strategies, including targeted agents with favorable tolerability profiles, must be balanced against surgical timing, perioperative morbidity, and the overarching priority of achieving optimal local control [103,105].

### 7.3. Emerging Biomarkers for Monitoring and Resistance

Emerging technologies for tumor monitoring and resistance detection are being explored in LPS. Mutational profiling of cell-free circulating DNA offers a non-invasive approach to tumor detection in MLPS, and individualized mini-panel sequencing of circulating tumor DNA allows tumor monitoring in complex karyotype sarcomas [109,110,111]. Circulating tumor DNA can also be used to monitor tumor treatment response by repeatedly analyzing blood samples during therapy, potentially helping guide optimal, individualized treatment [109,112]. While these technologies show promise in MLPS and other sarcoma subtypes, further validation is needed before routine clinical implementation in WDLPS/DDLPS, where genomic complexity and tumor biology may pose additional challenges.

**Figure 2:**
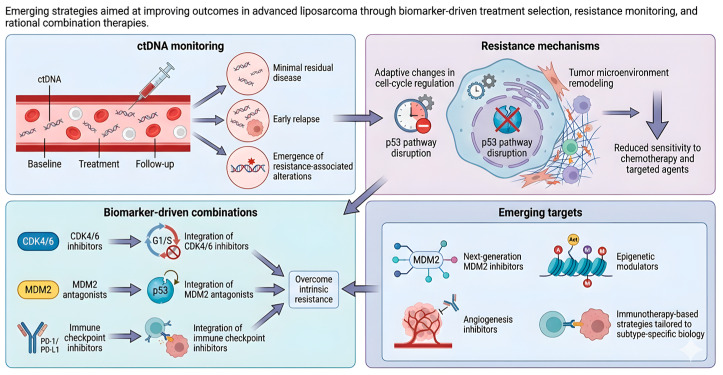
Future directions in systemic therapy for advanced liposarcoma. Created by the authors using Gemini (Google).

Adaptive resistance to cell-cycle and p53 pathway modulation, as well as tumor microenvironment-mediated immune escape, are major obstacles. Rational combinations (MDM2 inhibition + CDK4/6 inhibition; targeted therapy + immunotherapy; cellular therapy + checkpoint blockade) require careful toxicity management and early pharmacodynamic readouts to identify mechanisms of escape and guide treatment adaptation. These future directions, spanning ctDNA-based monitoring, resistance mechanisms, biomarker-driven combinations, and emerging therapeutic targets, are summarized in Figure 2.

### 7.4. Patient-Centered Outcomes and Treatment Sequencing

A systematic review of randomized clinical trials for advanced soft tissue sarcoma found that only 35% of trials included patient-reported outcomes (PROs), and none used PROs as primary endpoints, highlighting the need for improved and more consistent PRO reporting to inform patient care [113,114]. Quality of life assessment is particularly crucial in advanced sarcoma where treatment aims to balance efficacy with tolerability [113,115]. Given the chronicity of advanced WDLPS/DDLPS in many patients and the emerging availability of multiple treatment options, trials should incorporate quality-of-life and functional endpoints in addition to traditional survival outcomes to better inform evidence-based sequencing relative to chemotherapy and approved agents (eribulin, trabectedin, selinexor).

## 8. Conclusions

LPS management is undergoing a fundamental transformation from empirical cytotoxic therapy toward biology-driven, subtype-specific interventions. While complete surgical resection remains the cornerstone for localized disease, recent advances in targeted and cellular therapies have expanded options for advanced and metastatic disease. In WDLPS/DDLPS, characterized by near-universal MDM2 and CDK4 amplifications, second-generation MDM2–p53 antagonists such as brigimadlin show encouraging activity in ongoing phase II/III trials, CDK4/6 inhibitors demonstrate meaningful clinical benefit with manageable toxicity, and XPO1 inhibition with selinexor has achieved regulatory milestones despite modest efficacy. Perhaps most transformative has been the emergence of antigen-directed cellular therapy in MLPS: the 2024 FDA approval of afamitresgene autoleucel for synovial sarcoma and the 2025 breakthrough therapy designation for letetresgene autoleucel in MLPS represent paradigm shifts, validating TCR-engineered T cells as a viable strategy for tumors with defined antigenic targets. Immune checkpoint inhibitor combinations have demonstrated select activity in DDLPS, though predictive biomarkers remain elusive.

Critical to realizing the full potential of these advances is the integration of standardized molecular profiling into routine practice—including MDM2/CDK4 amplification testing for WDLPS/DDLPS and fusion detection with HLA typing for MLPS —to enable precision matching of patients to therapies. Substantial challenges remain: many targeted agents achieve stable disease but limited objective responses, combination approaches frequently encounter dose-limiting toxicities, and access to cellular therapies remains constrained by manufacturing complexity and cost. With continued collaboration across international sarcoma networks, rigorous integration of translational endpoints, and commitment to patient-centered outcomes, the emerging therapies reviewed here hold promise to meaningfully improve survival and quality of life for patients across all major LPS subtypes, marking a transition from a one-size-fits-all paradigm to molecularly informed, histology-tailored precision medicine.

## Figures and Tables

**Table 1:** Molecular characteristics, biomarkers, and systemic treatment strategies by liposarcoma subtype.

Liposarcoma Subtype	Key Molecular Features	Representative Biomarkers	Systemic Therapy Sensitivity	Common Systemic Therapy Options	Emerging/Investigational Approaches
**Well-differentiated/Dedifferentiated (WD/DDLPS)**	MDM2 and CDK4 amplification (12q13–15); low tumor mutational burden; relatively stable genome in WD, increased complexity in DDLPS	MDM2, CDK4 amplification (FISH/IHC); usually TP53 wild-type	Modest sensitivity to cytotoxic chemotherapy; relative chemoresistance compared with MLPS	Anthracycline-based chemotherapy ± ifosfamide (1 L); eribulin or trabectedin after anthracycline failure	MDM2 inhibitors (milademetan, brigimadlin); CDK4/6 inhibitors (palbociclib, abemaciclib); MDM2 + CDK4/6 combinations; XPO1 inhibition (selinexor); rational immunotherapy combinations
**Myxoid/Round cell (MLPS)**	Pathognomonic FUS::DDIT3 (or rarely EWSR1::DDIT3) fusion; translocation-driven transcriptional dysregulation; low MDM2 amplification	DDIT3 rearrangement; absence of MDM2 amplification	Highly sensitive to chemotherapy and trabectedin; relatively radiosensitive	Anthracycline ± ifosfamide; trabectedin; eribulin	NY-ESO-1-directed TCR-T therapy; immune checkpoint combinations; PI3K–AKT–mTOR pathway targeting; differentiation-based strategies
**Pleomorphic LPS (PLPS)**	Complex karyotype; frequent TP53 and RB1 alterations; high genomic instability	Lack of MDM2 and DDIT3 alterations	Variable but generally limited response to systemic therapy	Anthracycline-based chemotherapy; gemcitabine-based regimens; eribulin; trabectedin	Anti-angiogenic agents; immunotherapy in selected cases; clinical trial enrollment

**Table 2:** Pivotal Trials Supporting Current Standard Systemic Therapies in Advanced Liposarcoma.

Trial/Study	Phase	Population/Setting	Treatment Arms	Primary Endpoint	Key Efficacy Results (Selected)	Reference
EORTC 62012-Judson et al. (Dox + Ifos vs. Dox)	III	Advanced/metastatic or unresectable STS (incl. LPS), 1 L	Doxorubicin vs. Doxorubicin + Ifosfamide	Overall survival	OS: 12.8 vs. 14.3 mo (HR 0.83; *p* = 0.076); PFS: 4.6 vs. 7.4 mo (HR 0.74; *p* = 0.003); ORR: 14% vs. 26% (*p* < 0.0006); G3–4 febrile neutropenia: 13% vs. 46%	[13]
SAR-3007- Demetri et al. (Trabectedin vs. Dacarbazine)	III	Advanced LPS/LMS after anthracycline	Trabectedin vs. Dacarbazine	Overall survival	Median PFS: 4.2 vs. 1.5 mo (HR 0.55; *p* < 0.001); OS not significantly different in primary analysis	[19]
Schöffski et al. (Eribulin vs. Dacarbazine)	III	Advanced LPS/LMS after ≥2 prior regimens	Eribulin vs. Dacarbazine	Overall survival	Overall OS: 13.5 vs. 11.5 mo (HR 0.77; *p* = 0.0169); LPS subgroup OS: 15.6 vs. 8.4 mo (HR 0.51; *p* < 0.001); PFS: 2.9 vs. 1.7 mo (HR 0.52; *p* = 0.0015)	[25]

**Table 3:** Selected Clinical Trials of Investigational and Emerging Therapies in Liposarcoma.

Trial/Study	Phase	Subtype/Population	Regimen	Primary Endpoint	Key Efficacy Signal (Selected)	Reference
Palbociclib (Dickson et al.)	II (single-arm)	Advanced WD/DDLPS	Palbociclib 125 mg PO daily (21/28 days)	12-week PFS rate	12-week PFS ~50%; 1 CR >2 years; disease stabilization predominant	[46]
Abemaciclib (Phase II)	II (single-arm)	Progressive DDLPS	Abemaciclib (continuous dosing)	12-week PFS rate	12-week PFS 76%; median PFS ~30 weeks (~7 months)	[47]
SARC041 (NCT04967521)	III	Advanced DDLPS	Abemaciclib vs. placebo	PFS	Ongoing randomized study (~108 evaluable)	[48,49]
Palbociclib + Retifanlimab (NCT04438824)	II	Recurrent/unresectable/metastatic DDLPS	Palbociclib + anti-PD-1	ORR (RECIST 1.1)	≥4 confirmed responses required to reject null ORR 5% and support ORR 25% (ongoing)	[50]
Siremadlin + Ribociclib	Ib	Advanced WD/DDLPS	MDM2 inhibitor + CDK4/6 inhibitor	Safety/RP2D	Limited activity; dose-limiting hematologic toxicity	[51]
SEAL (NCT02606461) − Selinexor	II/III	Advanced, previously treated DDLPS	Selinexor vs. placebo	PFS	Median PFS: 2.8 vs. 2.1 mo (HR 0.70; *p* = 0.0228); no OS difference	[52]
Selinexor + Doxorubicin	Ib	Advanced STS (incl. DDLPS)	Selinexor + doxorubicin	Safety/ORR	ORR 21%; median PFS 5.5 mo; notable hematologic toxicity	[53]
Brigimadlin (BI 907828)	Ia/Ib	Advanced DDLPS	Brigimadlin 45 mg Q3W	Safety/activity	ORR 18.6%; median PFS 8.1 mo; DCR 88.4%	[40]
Brightline-1 (NCT05218499)	II/III	1 L advanced DDLPS	Brigimadlin vs. doxorubicin	PFS/OS	Ongoing comparative study	[41]
MANTRA (Milademetan vs. Trabectedin)	III	Advanced DDLPS	Milademetan vs. trabectedin	PFS	Median PFS 3.6 months (Milademetan) vs. 2.2 months (trabectedin)	[37]
IGNYTE-ESO (lete-cel)	II (pivotal)	Advanced MRCLS (NY-ESO-1+, HLA-A*02)	Letetresgene autoleucel (TCR-T)	ORR	MRCLS ORR 43% (13/30); median DoR 12.2 months	[54]
SPEARHEAD-1	II	HLA-A*02–positive, MAGE-A4–positive advanced synovial sarcoma (majority); small MRCLS cohort included	Afamitresgene autoleucel (single infusion following lymphodepletion with fludarabine + cyclophosphamide)	ORR	Synovial sarcoma cohort: ORR 43% (19/44); median duration of response ~6 months; manageable cytokine release syndrome	[55]

## Data Availability

No new data were created or analyzed in this study. Data sharing is not applicable to this article.
